# A Within-Subject Comparison of Face-to-Face and Telemedicine Screening Using the Timed Water Swallow Test (TWST) and the Test of Mastication and Swallowing of Solids (TOMASS)

**DOI:** 10.1007/s00455-022-10490-w

**Published:** 2022-07-09

**Authors:** Fredrik Karlsson, Leo Lovric, Josephine Matthelié, Louise Brage, Patricia Hägglund

**Affiliations:** Department of Clinical Sciences, Speech-Language Pathology, Umea University, 90187 Umea, Sweden

**Keywords:** Dysphagia screening, Comparison of administration situations, Telemedicine

## Abstract

The Timed Water Swallow Test (TWST) and the Test of Mastication of Solids (TOMASS) are dysphagia screening procedures that have been shown to be reliably assessed from video. The reliability of the procedures performed over telemedicine has not previously been assessed. TWST and TOMASS outcomes in two situations (both face-to-face and over telemedicine) were compared for 48 participants (aged 60–90; 27 with clinical conditions and 21 older persons). Both testing situation and test performed order were randomized, and all assessment procedures were performed within 3 h of each other. The results indicated a high level of agreement between face-to-face and telemedicine screening outcomes for TWST and TOMASS, respectively. The assessments indicated an 83% and 76% agreement in classifications of individual participants as within or outside normal limits for the TWST and TOMASS for the two test situations. The TWST showed a balanced distribution in differing classification in telemedicine (0.16–0.19 error rates). The TOMASS procedure classified more participants as outside normal limits over telemedicine compared to face-to-face administration. Agreement in the observed number of swallows was substantially lower than other outcome measures, which is attributed to increased difficulty in observing this property over video. Most participants (60%) reported that they would prefer telemedicine over face-to-face assessments, and 90% viewed the procedure as more accessible than expected. All participants were satisfied with the telemedicine procedures. The results suggest that clinical assessment of dysphagia over telemedicine using the TWST and TOMASS are viable alternatives to face-to-face administration of the procedures.

## Introduction

A reliable assessment of a person's ability to safely and effectively swallow food and drink is essential for ensuring a patient’s continued well-being [1]. The golden standard for assessing these abilities is observing a patient’s swallowing function using videofluoroscopic or videoendoscopic techniques, which require specialized medical equipment that may not be available in a clinic. Swallowing assessments may be an aerosol-generating procedure when coughing is provoked [2–4] and the COVID-19 pandemic has highlighted the need for procedures that may be performed without close contact between patient and caregiver. The need of providing swallowing assessment over longer distances, however, was observed long before the onset of the pandemic to remove factors such as long traveling times as barriers preventing early detection of dysphagia [5].

Efforts have already been made to enable synchronous instrumental or clinical swallow assessments by experts over telemedicine [6, 7] or to be performed asynchronously from a video recording [8]. However, these approaches for bridging the distance between assessor and patient require infrastructure that may not be available in all contexts. Perlman et al. (2002) used a system where videoflouroscopic recordings of a swallowing evaluation were transferred to a remote assessor station to be reviewed by an expert assessor [6], which limits the applicability to situations where both a videoflouroscopic video station and trained professional is available near the patient. The secured transfer, storage, and management of video-recorded medical evaluations between computers at different sites may raise additional concerns and infrastructure requirements. The study by Morell et al. [7] utilised an auto-guided teleconsultation cart to enable the assessor-patient interaction needed for post-stroke dysphagia in a remote clinic. While shown by Morell et al. [7] to provide an assessment of swallowing comparable to that performed onsite, the substantial investment needed in each remote location may prevent applicability of the service model in clinics with large uptake areas. Dysphagia screening procedures offer the opportunity for efficient identification of cases with possible dysphagia without requiring physical contact [2] or the expert to be present onsite to administer the test [4]. Several previous investigations of telemedicine assessments of dysphagia have, however, acknowledged the utility of an additional person being present in the room to assist the patient with practical aspects not part of the swallowing assessment [6, 7, 9], such as filling a cup of water or repositioning a camera. The person onsite is likely simply an additional safeguard against unforeseen issues that may be addressed locally, but the frequency of events that require local intervention have not formally been evaluated. Regardless, dysphagia screening over telemedicine may help offset barriers of long travel times and disease control requirements while allowing detection of potentially health-threatening swallowing dysfunction in the patient if shown to reproduce the results of onsite evaluations.

The Timed Water Swallow Test (TWST) and the Test of Mastication and Swallowing of Solids (TOMASS) were developed to support clinical swallow assessment of patient’s ability for safe swallowing liquid (TWST, 150 mL of water) or solid textures (TOMASS, a cracker) [10–13]. The TWST and TOMASS are dysphagia screening procedures that may be used to identify potential swallowing dysfunctions for subsequent instrumental assessment. Borders et al. [9] recently evaluated the possibility of performing the complete TWST and TOMASS procedures and assessing them from a video recording directed by the clinician but set up locally by the patient. Their results indicated that the TWST procedure had good-to-excellent inter-rater reliability in assessments of all outcome measures (swallowing capacity, swallowing time, and the number of swallows). The agreement with face-to-face testing outcomes were not assessed. Similarly, the TOMASS procedure was indicated to be assessed with good-to-excellent inter-rater reliability for the number of masticatory (chewing) cycles, time used for ingesting the cracker, and signs of aspiration outcome measures over a video connection. The inter-rater reliability was found to be considerably lower for the observation of the number of swallows when ingesting a cracker. Hägglund et al. [14] have reported similar results. Further, similar clinical swallow assessment procedures have indicated good agreement between outcomes of face-to-face and telemedicine test situations [15, 16] across dysphagia severity levels [16]. Whether it can be concluded that screening procedures like the TWST and TOMASS will have comparable outcomes when performed face-to-face and over telemedicine have, however, not been evaluated.

This study aimed to assess whether TWST and TOMASS administered fully over telemedicine could be considered a reliable alternative in cases when face-to-face administration is not practical or not advisable. Evaluations of clinical outcomes within the same participant morphology and in independent sessions were performed to reduce the risk inflating agreement by information transfer between sessions, a risk associated with simultaneous face-to-face and telemedicine testing highlighted by Ward et al. [17]. The secondary aim was to investigate which TWST and TOMASS outcome measures may have stronger disagreement between face-to-face and telemedicine administration.

## Method

This study is part of a research project that has been reviewed and approved by the National Ethical Review Authority (Case number 2020–04817).

### Participants

Forty-eight individuals aged 60–90 years old (27 men, average age = 75.2; 21 women, average age = 75.3) with self-perceived swallowing problems, a diagnosed dysphagia, or which may be suspected to show sub-clinical reductions in mastication and swallowing function due to increased age were recruited for the study. An overview of the participants is presented in Table 1. Recruitment was conducted in connection with the participants’ evaluation of swallowing function and from older friends and family of clinical staff. Persons with severe dysphagia preventing safe testing and who could not give informed consent were excluded from participation. One participant was excluded due to failure to complete the screen recording of telemedicine testing.

**Table 1:** An overview of the included participants and their conditions when tested

Participant condition	Men	Women
Stroke	11	11
Parkinson’s disease	2	
Huntington’s disease	1	
Cadasil		1
Hereditary spastic paraplegia	1	
Older persons without a diagnosed condition	12	9
Total number of participants	27	21

### Data collection

All participants were tested in two test situations: face-to-face and over telemedicine and using TWST and TOMASS in each test situation. The participants were randomized using two rolls of dice into (1) telemedicine or face-to-face first test situation order and (2) TWST or TOMASS first test order. The testing was performed by two assessors (authors LL and JM) who had received formal instruction in the TWST and TOMASS procedures as part of their SLP training. In the face-to-face test situation, the assessor administered the TWST and TOMASS according to the normal test procedure. In the telemedicine test situation, the assessor directed the procedure over the remote connection, and an additional person was available in the room to help with technical issues and when manual intervention was required [18]. The telemedicine testing used two video platforms with dedicated installations for clinical use were used in the telemedicine assessments (Plexip and Cisco Meeting, 768 × 448 pixels minimum video resolution and an 80 Kbits/s audio transfer speed). Forty-seven of the participants completed the face-to-face and telemedicine assessments within one hour of each other; one participant had approximately three hours delay between assessments. The data collection took approximately 25 min per participant to perform.

Prior to any testing, the participants ingested a teaspoon of water to ensure that there were no overt signs of aspiration. In the TWST procedure, the participants were asked first to produce a long /a:/ and then to ingest 150 mL of water as quickly but as comfortably as possible [10, 12]. The participants were also instructed to produce a long /a:/ after completing the ingestion to afford observation of gurgly (or wet) voice after testing. If the participant required a break in the procedure, the participant was asked to make a new /a:/ and then restart the assessment. The total time required for water ingestion was defined as the moment when the glass of water touched the lips to when the participant started to produce the /a:/. In cases where the participants forgot to produce an /a:/ directly after intake, the end time was defined as the moment when the larynx returned to a resting position. In the telemedicine test situation, the additional person in the room measured any residual water using a decilitre measurement and a plastic syringe with an mL scale. The swallowing time was determined online by the SLP instructing the patient (either face-to-face or over the telemedicine connection).

In the TOMASS assessment procedure, the participants were asked to ingest a TUC cracker as quickly but comfortably as possible and to produce a long /a:/ directly after to mark the end of ingestion [13, 14]. The swallowing time was measured from when the biscuit touched the lips to when the participant produced the long /a:/. The assessor observed the number of chewing cycles from the rotational movements of the jaw and the number of swallows by observing the raising of the larynx.

Upon completion of both the face-to-face and telemedicine assessments using both tests (TWST and TOMASS), the participants were asked to give their opinion of the test situations using two questions and one statement: “Do you feel satisfied with the assessment via telemedicine?” (yes/no), “The telemedicine assessment was easier than I expected” (affirm/disagree), and “Would you prefer an assessment via telemedicine over an assessment at the hospital clinic?” (yes/no).

All assessments were video recorded to afford assessment of inter-rater reliability. The participant was filmed from the tip of the nose down to the collarbone using the computer screen recording facilities (telemedicine assessments), the computer’s camera, or a mobile phone (in face-to-face administration). The inter-rater agreements were estimated from three independent raters’ (authors LL, JM, and LB) observation of TWST and TOMASS outcome measures from the video recording.

### Statistical Analysis

The number of chewing cycles (TOMASS), the number of swallows, and time taken to complete the task (TWST and TOMASS) obtained when assessed over telemedicine were compared to the standard clinical procedure using Bland & Altman’s methodology for comparison of methods [19] and Kendall’s rank correlation coefficient (Kendall’s τ). The presence of wet/gurgly voice was assessed using χ^2^ testing. Inter-rater agreements were assessed across three independent raters using Fleiss’ κ (observations of wet/gurgly voice and coughing) and Intraclass correlation coefficients, two-way random effects, absolute agreement, (ICC(2,1) [20]) for all other measures.

The clinical outcome for each participant (outside or within normal limits) when screened using TWST and TOMASS face-to-face and over telemedicine were further compared using percent agreement and False Positive Rate and False Negative Rate. For TWST, the swallowing capacity was computed as (150 mL—the observe residual liquid)/the swallowing time; a < 10 mL/s swallowing capacity was used as an indicator of dysphagia [10]. For the TOMASS, pooled means and standard deviations for swallowing time, number of chewing cycles, or the number of swallows were computed from previously published age and sex-stratified norms for the TUC cracker [13, 14] (Table 2). A value of 1.5 standard deviations above the derived mean was used as the cut-off for outside and within normal limits [21], and the participants were considered outside normal limits if above the cut-off in at least one TOMASS measure.

**Table 2:** Reference TOMASS outcome measures computed from the pooled age and sex-stratified norms for the TUC cracker [13, 14] of relevance for this study’s participants

Sex	Age range	N	Number of bites		Number of cycles		Number of swallows		Swallowing time	
			Mean ± SD	Cut-off	Mean ± SD	Cut-off	Mean ± SD	Cut-off	Mean ± SD	Cut-off
Males	20–39	29	2.4 ± 1.3	4.3	32.7 ± 12.2	51.0	1.6 ± 0.7	2.6	27.2 ± 10.2	42.4
	40–59	25	2.7 ± 1.1	4.3	36.3 ± 14.3	57.8	2 ± 1.1	3.6	31 ± 10.6	47.0
	60–79	29	3 ± 1.3	5.0	49.4 ± 18.4	77.0	1.4 ± 0.6	2.2	31.6 ± 10.1	46.7
	≥ 80	26	3 ± 1.3	5.0	34 ± 11.0	50.5	1.9 ± 0.9	3.3	37.6 ± 13.2	57.3
Females	20–39	29	3.2 ± 1.4	5.2	33.9 ± 9.7	48.4	1.7 ± 0.8	2.9	28.9 ± 7.8	40.5
	41–59	30	3.2 ± 1.2	5.0	42.6 ± 16	66.7	2 ± 1.3	4.0	31.6 ± 9.7	46.2
	61–79	35	3.7 ± 1.5	6.0	51.4 ± 16.7	76.5	2 ± 1.4	4.1	42.4 ± 14.9	64.7
	≥ 80	33	3.9 ± 1.2	5.7	1.6 ± 0.7	2.6	2.5 ± 1.1	4.2	51 ± 14.4	72.6

The value indicating 1.5 standard deviations above the pooled mean used as the cut-off for outside normal limits [21] is also indicated

## Results

The randomization of procedure order resulted in 30 of the 47 participants being assessed in telemedicine initially and then in the face-to-face situation. The observed agreement in clinical outcomes and clinical outcome measures between face-to-face and in telemedicine are presented below for TWST and TOMASS separately.

### The Timed Water Swallow Test (TWST)

The agreements in TWST screening outcomes (outside normal limits or within normal limits) of face-to-face and telemedicine assessments are presented as confusion matrices in Table 3. The percent exact agreement between the two test situations for the TWST was 76%. As indicated in Table 3, 16% of the participants that were identified as impaired in the face-to-face TWST screening were not identified as such in the telemedicine screening (False Negative Rate). The proportion of participants identified as impaired in telemedicine screening but not in the face-to-face (False Positive Rate) was 19%.

**Table 3:** The agreement in clinical screening outcomes of the Timed Water Swallow Test (TWST) and the Test of Mastication of Solids (TOMASS) for individual participants when assessed face-to-face and over telemedicine

Face-to-face screening (TWST/TOMASS)	Telemedicine screening			
	TWST		TOMASS	
	Outside normal limits^a^	Within normal limits	Outside normal limits^b^	Within normal limits
Outside normal limits	22	5	17	3
Within normal limits	3	16	8	17
Agreement		83%		76%
False positive rate		0.16		0.32
False negative rate		0.19		0.15

The fraction of missed and erroneously detected impairments are indicated by False Negative and False Positive Rates, respectively

^a^Participants were considered impaired if their swallowing capacity was below 10 mL/s

^b^Participants were considered impaired if their performances were above 1.5 standard deviations from the average score in healthy controls in any of the parameters swallowing time, number of chewing cycles, or number of swallows

**Fig. 1:**
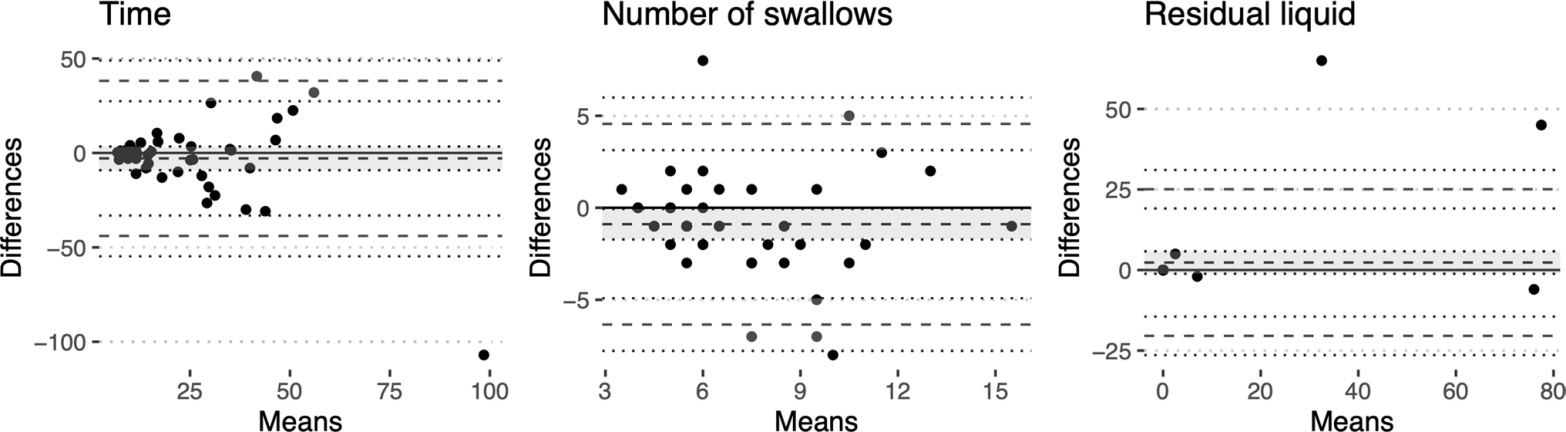
Bland–Altman plots for the assessment of time taken to swallow 150 mL of water (left), the number of swallows used (middle), and residual liquid (right) for Timed Water Swallow Test (TWST) in telemedicine compared to face-to-face administration. The horizontal axis shows the average of the two performance scores. The vertical axis shows the difference between the score obtained in telemedicine compared to face-to-face administration. The solid lines indicate no differences, and the dashed lines indicate the mean difference and the upper and lower 95% limits of agreement. Confidence intervals for the upper and lower limits of agreements are indicated by dotted lines, and the confidence interval for the mean is also shaded for clarity

The correlation between TWST outcome measures (number of swallows, total swallowing time, observation of gurgly voice, and of coughing) in face-to-face and telemedicine test situations is indicated in Table 4. The correlations in the number of swallows and total time used were observed to be significant (*τ* = 0.46, *p* < 0.001; *τ* = 0.58, *p* < 0.001). The Bland–Altman plots (Fig. 1) indicate that the zero-difference line was within the confidence interval of the mean difference between assessments for swallowing time but not for the number of swallows. The results showed a 78% agreement in whether a voice quality change following TWST was observed between assessments (χ^2^ = 55, *p* < 0.001). The observations of coughing showed no significant agreement (χ^2^ = 0.94, *p* = 0.33).

**Table 4:** The level of agreement between Timed Water Swallow Test (TWST) and Test of Mastication of Solids (TOMASS) outcome measures when assessed face-to-face and over telemedicine

Instrument	Measure	Agreement
TWST	Time	τ = 0.58, p < 0.001
	Swallows	τ = 0.46, p < 0.001
	Residual liquid	τ = 0.76, p < 0.001
	Gurgly voice	χ^2^ = 55, p < 0.001
	Cough	χ^2^ = 0.94, p = 0.33
TOMASS	Time	τ = 0.67, p < 0.001
	Swallows	τ = 0.15, p = 0.237
	Cycles	τ = 0.62, p < 0.001

The inter-rater agreements of the three raters observing TWST outcome measures from video recordings of the procedure are presented in Table 5. The intraclass correlations (two-way random, single measure, absolute agreement) of test outcomes were in the excellent range (> 0.90) [20] for number of swallows, time, and swallowing capacity. The observations of coughing and gurgly voice reached fair to moderate agreement when evaluated using Fleiss’ κ [22].

**Table 5:** The inter-rater reliability of outcome measures (Intraclass correlation coefficients, ICC, and Fleiss) of Timed Water Swallow Test (TWST) and Test of Mastication of Solids (TOMASS) when assessed from video, with confidence intervals

Dysphagia screening	Outcome measure	Inter-rater agreement	Failure to assess
TWST	Number of swallows	ICC(2,1) = 0.93 (0.86–0.96), p < 0.001	31%, κ = 0.84, p < 0.001
	Time	ICC(2,1) = 0.99 (0.98–0.99), p < 0.001	31%, κ = 0.88, p < 0.001
	Swallowing capacity	ICC(2,1) = 0.97 (0.94–0.98), p < 0.001	31%, κ = 0.85, p < 0.001
	Gurgly voice	κ = 0.40, p = 0.06	32%, κ = 0.93, p < 0.001
	Coughing	κ = 0.49, p = 0.02	32%, κ = 0.94, p < 0.001
TOMASS	Number of chewing cycles	ICC(2,1) = 0.89 (0.73–0.95), p < 0.001	26%, κ = 0.62, p < 0.001
	Number of swallows	ICC(2,1) = 0.75 (0.43–0.88), p < 0.001	26%, κ = 0.65, p < 0.001
	Time	ICC(2,1) = 0.99 (0.98–1.00), p < 0.001	28%, κ = 0.75, p < 0.001

The reliability of rater’s judgments of the quantity not being possible to assess is also indicated

### The Test of Mastication of Solids (TOMASS)

The agreements in TOMASS screening outcomes (outside normal limits or within normal limits) of face-to-face and telemedicine assessments are presented as confusion matrices in Table 3. The percent exact agreement between the two test situations for the TOMASS was 83%. The comparison of TOMASS assessments indicated an elevated False Positive Rate (0.32) compared to all other rates of differential screening outcomes (0.15–0.19) in Table 3. The higher False Positive Rate value for the TOMASS assessment was caused by over-classification as impaired based on the number of swallows over telemedicine (False Positive Rate = 0.56), with an additional contribution of the number of chewing cycles (False Positive Rate = 0.33). The False Positive Rate of TOMASS swallowing time was not elevated compared to the other comparisons (False Positive Rate = 0.18).

**Fig. 2:**
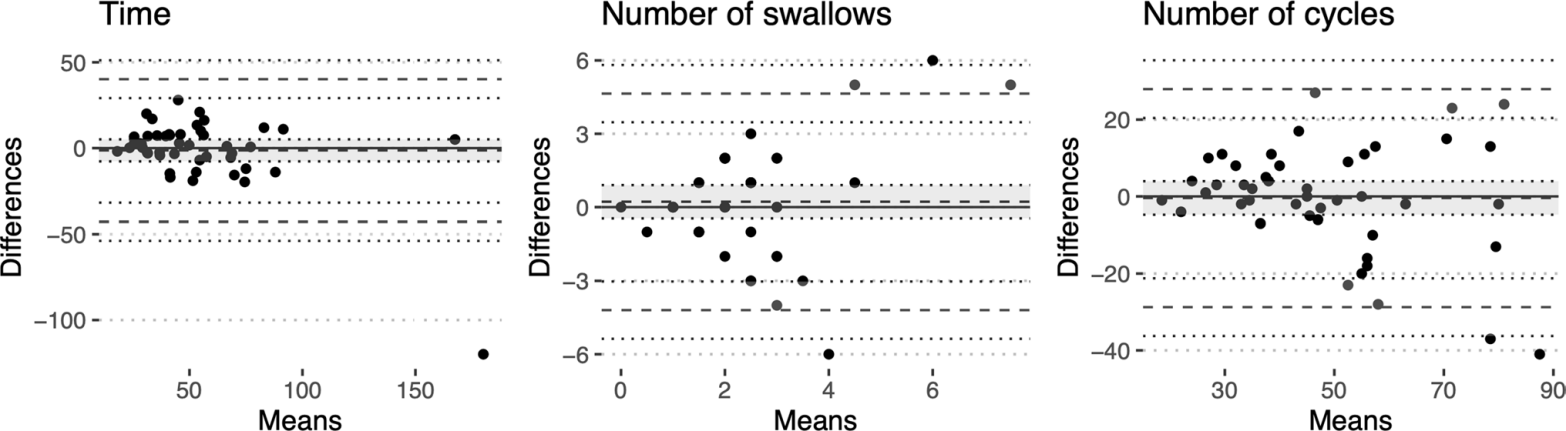
Bland–Altman plots for the assessment of time taken to swallow a cracker (left), the number of swallows (middle), and the number of chewing cycles (right) in Test of Mastication of Solids (TOMASS) in telemedicine compared to face-to-face administration. The horizontal axis shows the average of the two performance scores. The vertical axis shows the difference between the score obtained in telemedicine compared to face-to-face administration. The solid lines indicate no differences, and the dashed lines indicate the mean difference and the upper and lower 95% limits of agreement. Confidence intervals for the upper and lower limits of agreements are indicated by dotted lines, and the confidence interval for the mean is also shaded for clarity

The correlation between TOMASS outcome measures (total swallowing time, number of swallows, and number of chewing cycles) in face-to-face and telemedicine test situations is indicated in Table 4. Table 2 further indicates the correlation between outcome measures obtained for the participants in face-to-face and telemedicine situations for the TOMASS. Figure 2 presents the difference between each patient’s assessments made the same day using Bland–Altman plots [19]. The solid lines indicated no difference in scores in all sub-figures. The confidence region of the mean difference between assessments is indicated using dashed lines and gray shading. A considerable spread in differences in the number of swallows is observed in Fig. 2, which agrees with the low correlation statistic for the measure (Table 2).

The inter-rater agreements of the three raters observing TOMASS outcome measures from video recordings of the procedure are presented in Table 5. The intraclass correlations (two-way random, single measure, absolute agreement) for swallowing time was in the excellent range (> 0.90), and the number of chewing cycles and number of swallows were good (0.75–0.90) reliability range [20]. The agreement between raters on whether a measurement or observation could not be made were all > 0.60, indicating a substantial (or better) agreement [22].

### Participants’ Satisfaction with Telemedicine Screening

In the questionnaire following up on the participants' subjective view of the testing procedures, all participants answered Yes to the question “Do you feel satisfied with the assessment via telemedicine?”. Forty-one participants (90%) reported that “The telemedicine assessment was easier than I expected”. Twenty-eight participants (60%) answered Yes to the question, “Would you prefer an assessment via telemedicine over an assessment at the hospital clinic?”.

## Discussion

This study aimed at estimating the reliability of results from the screening tools TWST and TOMASS over telemedicine. The TWST and TOMASS were assessed for each participant in separate face-to-face and telemedicine test situations within one hour of each other (one participant had a three hour time gap), with testing orders randomized for each participant. The study constitutes the first direct comparison of TWST and TOMASS outcomes in face-to-face and telemedicine test situations within the same patient morphology. The separate, rather than simultaneous, sessions for the two test situations provided a conservative estimate of agreement between outcomes due to incorporating also test–retest and inter-observed variabilities. However, the independence of assessments achieved by using independent assessors negated the risk of artificially increased agreement in outcomes due to transfer between assessments that have been acknowledged in simultaneous evaluations [17].

The results from comparison of clinical screening outcomes, correlation analysis and visual evaluation using the Bland and Altmann methodology for comparing methods indicated a good correspondence between TWST and TOMASS assessed face-to-face and over telemedicine. In addition, the participants reported a high level of satisfaction with the telemedicine screening procedure, which is in line with previous research [23]. The TWST showed a balanced distribution in differing classification in telemedicine. The TOMASS procedure classified more participants as outside normal limits over telemedicine compared to face-to-face administration. The TWST showed a balanced distribution in differing classification in telemedicine (0.16–0.19 error rates). The TOMASS procedure classified more participants as outside normal limits over telemedicine compared to face-to-face administration. The number of observed swallows outcome measures of both TWST and TOMASS was, however, more difficult to assess reliably over telemedicine, which has been observed also in the evaluation of inter-rater reliability of assessments over telemedicine reported on by Borders et al. [9]. The number of swallows observed during the TOMASS was the primary contributor to the elevated classification of participants as impaired in telemedicine administration of the screening procedure compared to the face-to-face situation. Further, the inter-rater agreement from video was the lowest for the number of swallows in the TOMASS. While adjustment of the 1.5 SD cut-off used here as well as Heul et al. [21] may improve agreement in clinical classification outcomes, the results overall suggest that the number of swallows is the most difficult measure from a video transfer of a screening procedure and that TOMASS may be particularly affected. As noted by Borders et al. [9], the cause of the reduced agreement in the number of swallows outcome measure between screening situations may be related to telemedicine specific barriers such as suboptimal viewing angle or obstruction of the mouth while chewing. In face-to-face testing, the observer is freer to adjust their viewing angle to enhance their ability to make observations. However, since we, like Borders et al. [9], sought a high ecological validity in our comparison of the two test situations, a heterogeneous sample of participants with several different underlying causes with an increased risk of dysphagia were investigated in the current study. Borders et al. notes that the underlying disease may often cause, for instance, extraneous lingual movements, which may be mistaken for masticatory cycles [9] and reduce the reliability of observing true swallowing movements. Whether this issue may be mitigated using a better camera placement or other adjustments to the procedure does, however, require a separate investigation.

In this study, the TWST and TOMASS performance of participants were assessed in the same room and over telemedicine and after the fact (asynchronously) from a video recording. In a recent review, Miles et al. [2] concluded based on previous reports [17, 25–27] that both asynchronous and synchronous dysphagia screening procedures and videofluoroscopic evaluations of dysphagia might be made with high reliability but did not evaluate agreement in individual outcome measures. Our results show a more substantial presence of failure to assess individual outcome measures from asynchronous observations of video than the synchronous counterpart. Synchronous assessment of a patient over telemedicine with a person in the room [9, 18] or in the form of patient-administered recording [9] offer the opportunity to communicate a failure to observe the outcome measure so that adjustments to the recording setup can be made. In our study, the assessor could direct what was transferred over the video to their preference and afford reliable assessment. In contrast, in a video-recorded (asynchronous) assessment, the video is adjusted to the needs of the director of the recording, which may not suit all observers. This study evaluated the agreement of two complete administrations of the TWST and TOMASS, and therefore involve a risk of transfer between performances. However, both TWST and TOMASS have been reported to have good test–retest properties [13, 28], and the randomization of administration order should have mitigated this effect. The use of a within-participant design instead allowed us to keep the anatomical structures and level of impairment consistent between screening sessions, which were perceived as a larger concern for this evaluation. Therefore, we argue, based on our results, that synchronous administration of the TWST and TOMASS procedures, but with an additional person in the room with the patient, is the preferable way to screen for dysphagia over telemedicine. The person in the room does not need to be a health care professional as instructions may be given over the audio/video connection.

## Conclusions

The Timed Water Swallow Test (TWST) and the Test of Mastication of Solids (TOMASS) showed a good level of agreement in outcomes when performed over telemedicine compared to face-to-face administration, except for the number of swallows which may be more difficult to assess. The participants reported a high level of satisfaction with the telemedicine administration of the screening procedures, and most would prefer telemedicine over face-to-face screening. Depending on cut-offs used, the TOMASS procedure may result in an increased rate of false positive screening outcomes over telemedicine compared to face-to-face administration, but at a level that is viewed as acceptable considering the substantial advantages of remote administration of the test procedure.
